# Editorial: From Trial and Error to Individualised Pharmacogenomics-Based Pharmacotherapy in Psychiatry

**DOI:** 10.3389/fphar.2021.725565

**Published:** 2021-09-22

**Authors:** R. van Westrhenen, M. Ingelman-Sundberg

**Affiliations:** ^1^ Parnassia Psychiatric Institute, Amsterdam, Netherlands; ^2^ Department of Psychiatry and Neuropsychology, Maastricht University, Maastricht, Netherlands; ^3^ Institute of Psychiatry, Psychology and Neuroscience (IoPPN) Kings College, London, United Kingdom; ^4^ Department of Physiology and Pharmacology, Karolinska Institute, Stockholm, Sweden

**Keywords:** pharmacogenomics, psychiatry, psychopharmacology, CYP enzymes, side effects

It has been reported that only 30% of patients use psychopharmacological treatment with perceived clinical success (SBU rapport Swedish Research Council, 2004; Walker et al., 2004), suggesting that the majority of psychiatric patients do not successfully respond to the drugs. The explanations include inefficacy or side effects, caused by e.g., altered rate of drug elimination or unfavorable drug-drug interactions due to polypharmacy. The side effects include dizziness, dry mouth, headache, nausea, weight gain, and decreased sexual desire. These side effects are often so severe that patients do not comply to or discontinue medication.

Novel drug regimens for depression and schizophrenia have not been developed for many years emphasizing the importance of optimizing treatment with the drugs at hand. Genetic variations, particularly in the genes *CYP2C19* and *CYP2D6*, have been important to explain elevated risk for side effects or for switching of antipsychotic or antidepressant medicines (Milosavljevic et al., 2021). Indeed, a higher frequency of subjects with an ultrarapid (UM) CYP2D6 phenotype has been observed in patients committing suicide indicating non-optimal dosing of antidepressants in these patients and elevated levels of antidepressant drugs in poor metabolizers (PMs) of CYP2D6 and CYP2C19 substrates suggest possibilities of therapeutic improvements by pre-emptive genotyping of such patients (Jukic et al., 2019; van Westrhenen et al., 2020; Islam et al., 2021).

Pharmacogenetic polymorphisms that influence the metabolism of drugs are common. The prevalence of PM status among Europeans is 3% for CYP2C19 and 8% are CYP2D6 PMs. Intermediate metabolizer (IM) status is much more common: among Europeans, 17% are CYP2C19 IM and 30% are CYP2D6 IM and these are important determinants of side effects and, thus, therapeutic success (Jukic et al., 2019; van Westrhenen et al., 2020). The translation of the genetic variations in these genes to firm recommendations to be used in the clinics represents is an important but also difficult task based on i) the need for concordance in opinions between different organizations and regulatory units regarding the importance of different pharmacogenomic biomarkers and ii) the problem of application of such advice in clinical practice due to the complexity of a real-life patient setting and unwillingness of the physicians to comply with such advice (Roberts, 2018). Both the Dutch Pharmacogenetic Working Group (DPWG, see https://www.pharmgkb.org/page/dpwg) and the Clinical Pharmacogenetics Implementation Consortium (CPIC) have formulated advice regarding genetically based drug treatment in psychiatry medicine (https://cpicpgx.org/guidelines/) (Bank et al., 2018; Swen et al., 2018) but there is a major problem in the consistency of recommended pharmacogenomic biomarkers between CPIC, FDA, EMA, and DPGW as described (Shekhani et al., 2020). Thus, of 54 drugs with an actionable gene–drug interaction in the CPIC and DPWG guidelines, <50% had actionable pharmacogenomic information in the labels in the SmPCs of EMA and the FDA (Shekhani et al., 2020). Only 18% of the cases were in agreement between CPIC, DPWG, FDA, and EMA. The consensus of actionable pharmacogenomic labels of 184 different gene–drug interactions between the FDA and EMA was only 54% (Shekhani et al., 2020).

However, recent data do indicate that preemptive genotyping for *CYP2C19* and *CYP2D6* has promise and the specific phenotypes based on genotyping are related to the observed pharmacokinetics of antipsychotic and antidepressant drugs and also to the likelihood of switching drugs during the treatment (Jukic et al., 2019; van Westrhenen et al., 2020; Carvalho Henriques et al., 2020; Islam et al., 2021). Furthermore, after evaluation of 1,159 studies Karamperis et al. found that only CYP2C19 and CYP2D6 drug gene associations did exhibit cost benefit in psychiatric pharmacogenomics (Karamperis et al., 2021).

One problem though concerning the current genotyping is the fact that within each phenotypically defined group there is a interindividual variability that has to be overcome in order to ensure a specific personalized drug treatment. Such variability is related to i) rare genetic variants (McInnes et al., 2021), ii) to the fact that each allele defined by the identified functional mutation harbors several different haplotypes as evidenced from analyses of CYP2C locus haplotypes and metabolism of SSRI antidepressants (Bråten et al., 2021) but iii) also by remote mutations as revealed from the influence of NFIB polymorphism on the level of clozapine levels (Løvsletten Smith et al., 2020). We do believe that future identification of novel haplotypes and implementation of new genetic loci will improve the predictability of genotyping and persuade clinicians being critical to genotyping, to indeed implement pharmacogenomics in psychiatry.

In this issue the role of CYP2C19 and CYP2D6 polymorphisms in psychiatry is reviewed based on different aspects as well as the pharmacogenomics of lithium treatment and also the European initiative for implementation of pharmacogenomics in psychiatry is described (https://www.psy-pgx.org/PSY-PGx).

Van Westrhenen et al. present a guideline on clinical implementation of pharmacogenetics in psychiatry. They advise that only for CYP2C19 and CYP2D6 there seems enough evidence from clinical prospective studies to perform genotyping in clinical psychiatric settings (Van Westrhenen et al.).

Just et al. focuses on the variability in CYP2D6 activity linked to adverse drug reactions (ADRs) in the CNS, a novel and potentially very important aspect and describes a link between drug-related CNS symptoms and CYP2D6 activity. Based on the ADRED study they describe that the CYP2D6 activity was positively associated with dizziness, but not with nonvigilance-related ADR symptom such as syncope or nausea. (Just et al.).

In the presentation by Molden and Jukic, data are presented from several different high power studies suggesting that the assigned activity scores of reduced function variant CYP2D6 alleles in current guidelines are not of sufficient precision and the authors state that it is thus important that the guidelines are updated to be valid in predicting individual dose requirements (Molden and Jukic).

Senner et al., delineate the mechanisms of action of lithium and summarize the results of genetic research on lithium response and side effects. They conclude that no confirmed genetic polymorphisms can predict lithium response but that there is a need for pharmacogenetic research regarding tolerability and anti-suicidal effects of lithium. *Frontiers | Selective Serotonin Reuptake Inhibitor Pharmaco-Omics: Mechanisms and Prediction | Pharmacology*, Senner et al.

Lastly, the recent Horizon 2020 PSY-PGx Project is presented where a non-industry linked multicenter worldwide pharmacogenetics study will be carried out in psychiatric patients using AI-based predictive models to provide more accurate personalized medication for more effective drug treatment in psychiatry [A New Intervention for Implementation of Pharmacogenetics in Psychiatry | PSY-PGx Project | H2020 | CORDIS | European Commission (europa.eu)]. Such randomized large clinical trials are important also in the future to define the impact and cost effectiveness of genotyping in psychiatry.
